# P24 Cefiderocol activity against a multidrug-resistant (MDR) strain of *Pseudomonas aeruginosa*: a case report

**DOI:** 10.1093/jacamr/dlac004.023

**Published:** 2022-02-16

**Authors:** Felicia H. Lim,, Corrine Ashton, David R. Jenkins

**Affiliations:** Department of Clinical Microbiology, University Hospitals of Leicester NHS Trust, UK; Pharmacy Department, University Hospitals of Leicester NHS Trust, UK; Department of Clinical Microbiology, University Hospitals of Leicester NHS Trust, UK

## Abstract

**Background:**

MDR Gram-negative bacteria such as *Pseudomonas aeruginosa* represent the highest priority for addressing global antibiotic resistance. Moreover, selecting an appropriate empirical antibiotic treatment for MDR strains is a challenge. Cefiderocol, a novel siderophore cephalosporin, has demonstrated activity against Gram-negative strains resistant to other available antibiotics.

**Patient case:**

A 58-year-old female was admitted on to the ICU with type 1 respiratory failure as a consequence of COVID-19 pneumonitis. During the course of her ICU admission she developed persistent bilateral empyema and pneumothorax which did not resolve despite the use of pleural drains. The empyema samples grew *P. aeruginosa*.

**Treatment course:**

The patient was treated with commonly used antibiotics for *P. aeruginosa*, including piperacillin/tazobactam, meropenem, ceftolozane/tazobactam and ciprofloxacin. However, repeated testing of empyema fluid and respiratory secretions identified an increasingly drug-resistant *P. aeruginosa* strain. Following left thoracotomy and pleural decortication, cefiderocol was approved for compassionate use and the patient was treated for 14 days. The patient experienced good resolution of clinical symptoms and C-reactive protein levels and was discharged to the ward after 105 days in the ICU. The patient was briefly readmitted to the ICU with worsening type 2 respiratory failure 2 weeks later. Ceftolozane/tazobactam and colistin treatment were recommenced as a bridging measure until cefiderocol became available in the hospital. Notably, the *P. aeruginosa* isolated remained susceptible to cefiderocol following the first course of treatment. Cefiderocol therapy (2 g t.i.d.) was recommenced for 14 days of ward based treatment. The patient demonstrated clinical and radiographic resolution of her infection and was eventually discharged home.

**Conclusions:**

This patient case describes a heavily pretreated female with a MDR *P. aeruginosa* strain successfully managed with cefiderocol and source control. Cefiderocol was prescribed for compassionate use for treatment of MDR *P. aeruginosa*. Treatment was well tolerated and led to good resolution of clinical symptoms. Cefiderocol may help address the global issue of MDR *P. aeruginosa*.

